# Antioxidant Tannins from Stem Bark and Fine Root of *Casuarina equisetifolia*

**DOI:** 10.3390/molecules15085658

**Published:** 2010-08-16

**Authors:** Shang-Ju Zhang, Yi-Ming Lin, Hai-Chao Zhou, Shu-Dong Wei, Guang-Hui Lin, Gong-Fu Ye

**Affiliations:** 1 Key Laboratory of the Ministry of Education for Coastal and Wetland Ecosystems, School of Life Sciences, Xiamen University, Xiamen 361005, China; 2 Fujian Academy of Forestry, Fuzhou 350012, China

**Keywords:** *Casuarina equisetifolia*, condensed tannins, MALDI-TOF MS, HPLC, antioxidant activity

## Abstract

Structures of condensed tannins from the stem bark and fine root of *Casuarina equisetifolia* were identified using MALDI-TOF MS and HPLC analyses. The condensed tannins from stem bark and fine root consist predominantly of procyanidin combined with prodelphinidin and propelargonidin, and epicatechin is the main extension unit. The condensed tannins had different polymer chain lengths, varying from trimers to tridecamer for stem bark and to pentadecamer for fine root. The antioxidant activities were measured by two models: 1,1-diphenyl-2- picrylhydrazyl (DPPH) radical scavenging activity and ferric reducing/ antioxidant power (FRAP). The condensed tannins extracted from *C. equisetifolia* showed very good DPPH radical scavenging activity and ferric reducing/ antioxidant power, suggesting that these extracts may be considered as new sources of natural antioxidants for food and nutraceutical products.

## 1. Introduction

Condensed tannins are a class of secondary metabolites with pronounced biological activities found in many plants [1]. Condensed tannins are formed of flavan-3-ol units, which are linked together through C4–C6 or C4–C8 bonds to oligomers and high molecular weight polymers [2,3,4,5]. The diversity of condensed tannins is given by the structural variability of the monomer units: different hydroxylation patterns of the aromatic rings A and B, different stereochemistry at the chiral centers C2 and C3, and the distinct location and stereochemistry of the interflavanoid bond [2,3,4,5]. 

Condensed tannins, a major group with antioxidant properties, and act against allergies, ulcers, tumours, platelet aggregation, cardiovascular diseases and can reduce the risk of cancer [6]. The bioactivity capacity of plant tannins is generally recognized to be largely dependent on their structure and particularly the degree of polymerization [7]. However, tannins are diverse compounds with great variation in structure and concentration within and among plant species. Due to the diversity and structural complexity of highly polymerized tannins, the analysis and characterization of condensed tannins is a difficult task, and less is known regarding structure-activity relationships [8,9]. Various techniques including NMR, acid-catalyzed depolymerization of the polymers in the presence of nucleophilic reagents, and MALDI-TOF MS have been used to characterize condensed tannins [10,11,12,13,14,15].

*Casuarina equisetifolia* is traditionally used as a medicinal plant [16,17]. The phenolic compounds from branchlets (leaf) and bark showed the significant antioxidant activity [17]. Therefore, this plant might be a good candidate for further development as a nutraceutical or for its antioxidant remedies. However, the structures of the condensed tannins from *C. equisetifolia* were rarely studied [18], and detailed information on the condensed tannins profiles, especially with respect to polymer chain length, chemical constitution of individual chains, and the sequential succession of monomer units in individual chains present in *C. equisetifolia* is currently lacking. In this study, contents of total phenolics and extractable condensed tannins of stem bark and fine root of *C. equisetifolia* were determined, and the structures of condensed tannins from stem bark and fine root were characterized by MALDI-TOF MS and HPLC analyses. The free radical scavenging capacities and ferric reducing power of condensed tannins from stem bark and fine root were also evaluated.

## 2. Results and Discussion

### 2.1. Content of Total Phenolics and Extractable Condensed Tannins

Total phenolic contents in the stem bark and fine root of *C. equisetifolia* were 110.83 ± 3.65 mg/g and 106.23 ± 11.28 mg/g, respectively (Table 1). Extractable condensed tannin contents in the stem bark and fine root of *C. equisetifolia* were 112.69 ± 6.67 mg/g and 116.33 ± 10.65 mg/g, respectively. Stem bark and fine root of *C. equisetifolia* thus have the same tannin levels (Table 1).

It is well-known that diets rich in fruit and vegetables are protective against cardiovascular disease and certain forms of cancer [19]. These protective effects have been attributed, in large part, to the antioxidants including plant phenolics such as the tannins [20]. Phenols are very important plant constituents because of their radical scavenging ability due to their hydroxyl groups. The phenolic content may contribute directly to the antioxidative action [21]. In the present study, the stem bark and fine root of *C. equisetifolia* show relatively high phenolics levels, which may be potential sources of natural antioxidants.

**Table 1:** Contents of total phenolics and extractable condensed tannins in stem bark and fine root of *C. equisetifolia* (n = 3).

Samples	Total phenolics *^a^*	Extractable condensed *^b^*
	(mg/g dry weight)	tannins (mg/g dry weight)
Stem bark	110.83 ± 3.65a	112.69 ± 6.67a
Fine root	106.23 ± 11.28a	116.33 ± 10.65a

*^a^* Using tannic acid as the standards; *^b^* Using respective purified tannins from stem bark and fine root as the standards. Different letters in the same column show significant differences from each other at *P* < 0.05 level.

### 2.2. MALDI-TOF MS Analysis

Many previous studies have shown that MALDI-TOF MS is a powerful technique for the characterization of both synthetic and natural polymers (including condensed tannins) [22,23,24,25,26]. In our study, according to the findings of Xiang *et al.* [27], the CS^+^ was used as the cationization reagent. This resulted in the best conditions for their MALDI-TOF analysis and a relatively simple MALDI-TOF spectrum.

**Figure 1:**
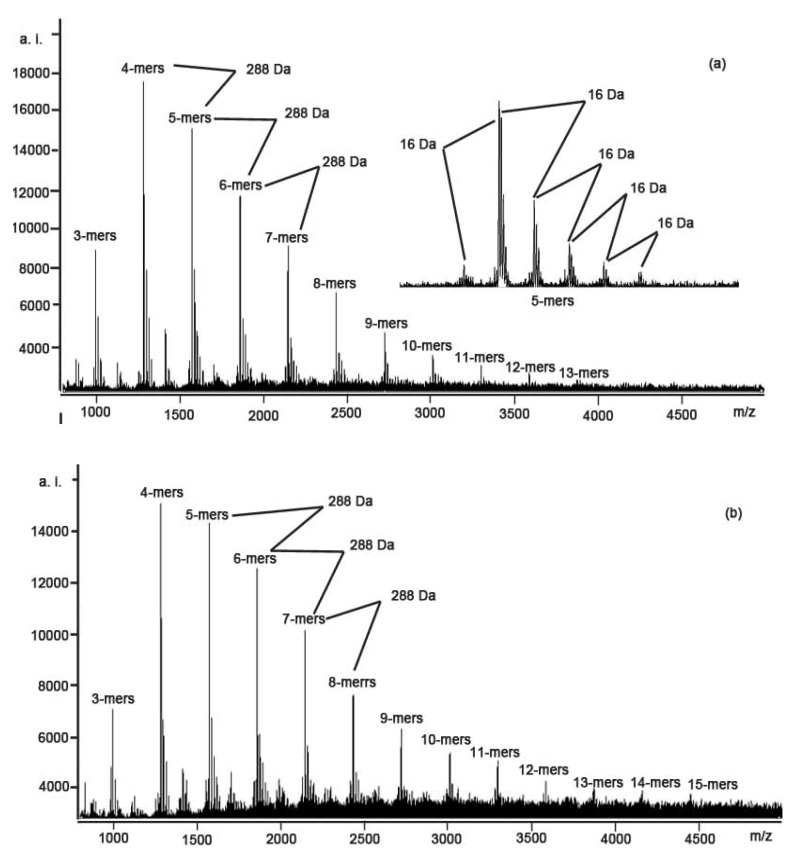
MALDI-TOF positive reflectron mode mass spectra of the condensed tannins from (a) stem bark and (b) fine root of *C. equisetifolia*.

Figure 1 shows the MALDI-TOF mass spectra of the polymeric condensed tannins mixture of the stem bark and fine root of *C. equisetifolia*, recorded as CS^+^ adducts in the positive ion reflectron mode. The polymeric character is reflected by the periodic peak series representing different chain lengths. The results indicate that condensed tannins isolated from the stem bark and fine root are characterized by mass spectra with a series of peaks with distances of 288 Da, corresponding to one catechin/ epicatechin monomer (Table 2). The condensed tannins from the stem bark and fine root of *C. equisetifolia* had different polymer chain length varying from trimers to tridecamer for stem bark and to pentadecamer for fine root.

In addition to the predicted homopolyflavan-3-ol mass series mentioned above, each DP had a subset of masses 16 Da higher were also detected, which can be explained by heteropolymers of repeating flavan-3-ol units containing an additional hydroxyl group at the position 5' of the B-ring. Each DP had a subset of masses 16 Da lower in the spectra of condensed tannins from stem bark and fine root (Figure 1 and Table 2). These masses indicated the polymer chains containing monomers with only one hydroxyl group (16 Da) at the aromatic ring B. Given the absolute masses corresponding to each peak, it was further suggested that the condensed tannins from stem bark and fine root contain procyanidin, prodelphindin and propelargonidin, both with the procyanidin dominating. The structures of condensed tannins from stem bark and fine roots of *C. equisetifolia* are successfully characterized using MALDI-TOF MS for the first time.

**Table 2:** Summary of peaks with the highest intensities in MALDI-TOF MS of the condensed tannins from stem bark and fine root of *C. equisetifolia.*

Polymer	n_1_	n_2_	n_3_	Calculated	Observed [M + Cs]^+^	
				[M + Cs]^+^	Stem bark	Fine root
Trimer	0	3	0	999	999.27	999.24
	1	2	0	983	983.11	983.25
	0	2	1	1015	1015.24	1015.25
	0	1	2	1031	1031.24	1031.24
	0	0	3	1047	1047.22	1046.83
Tetramer	0	4	0	1287	1287.30	1287.28
	1	3	0	1271	1271.31	1271.25
	0	3	1	1303	1303.30	1303.23
	0	2	2	1319	1319.29	1319.30
	0	1	3	1335	1335.49	1335.20
	0	0	4	1351	1351.39	1351.23
Pentamer	0	5	0	1575	1575.34	1575.30
	1	4	0	1559	1559.32	1559.30
	0	4	1	1591	1591.48	1591.33
	0	3	2	1607	1607.49	1607.32
	0	2	3	1623	1623.32	1623.27
	0	1	4	1639	1640.48	1639.30
Hexamer	0	6	0	1863	1863.34	1863.34
	1	5	0	1847	1847.37	1847.47
	0	5	1	1879	1879.36	1879.34
	0	4	2	1895	1895.39	1896.45
	0	3	3	1911	1911.34	--
	0	2	4	1927	1928.68	--
Heptamer	0	7	0	2151	2151.41	2152.36
	1	6	0	2135	2135.39	2135.51
	0	6	1	2167	2167.35	2167.36
	0	5	2	2183	2183.38	2183.31
	0	4	3	2199	2199.36	2200.33
	0	3	4	2215	2215.94	2215.49
Octamer	0	8	0	2439	2440.40	2439.48
	1	7	0	2423	2423.47	2423.51
	0	7	1	2455	2456.40	2456.28
	0	6	2	2471	2472.51	2471.52
	0	5	3	2487	2488.40	2489.49
	0	4	4	2503	2504.42	2503.56
Nonamer	0	9	0	2727	2728.39	2728.42
	1	8	0	2711	2712.39	2711.06
	0	8	1	2743	2744.39	2744.31
	0	7	2	2759	2760.38	2759.47
	0	6	3	2775	2775.42	2776.33
Decamer	0	10	0	3015	3016.44	3016.51
	1	9	0	2999	3001.36	3000.16
	0	9	1	3031	3032.92	3032.29
	0	8	2	3047	3048.48	3049.47
	0	7	3	3063	3064.37	--
	0	6	4	3079	3079.48	--
Undecamer	0	11	0	3303	3304.19	3305.48
	1	10	0	3287	--	3289.40
	0	10	1	3319	3320.75	3319.19
	0	9	2	3335	3335.95	3335.78
	0	8	3	3351	3351.45	--
Dodecamer	0	12	0	3591	3592.75	3592.98
	1	11	0	3575	--	3577.91
Tridecamer	0	13	0	3879	3880.24	3880.90
	0	12	1	3895	--	3896.31
Tetradecamer	0	14	0	4167	--	4169.66
	0	13	1	4183	--	4184.57
Pentadecamer	0	15	0	4455		4456.93

n_1_: Number of afzelechin/epiafzelechin unit; n_2_: Number of catechin/epicatechin unit; n_3_: Number of gallocatechin/epigallocatechin unit; “--” means no observed peaks corresponding to those calculated ones.

### 2.3. Thiolysis with Cysteamine Followed by RP-HPLC Analysis

**Figure 2:**
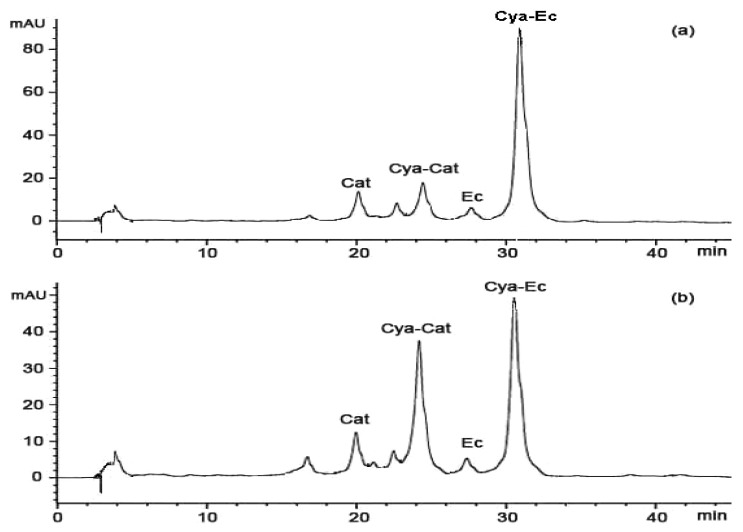
Reversed phase HPLC chromatograms of the condensed tannins from: (a) stem bark and (b) fine root degraded in the presence of cysteamine; Cat, (+)-catechin; EC, (-)-epicatechin; Cya-Cat, 4β-(2-aminoethylthio)catechin; Cya-EC, 4β-(2-aminoethylthio)-epicatechin.

To further investigate whether the condensed tannins from stem bark and fine root are composed of catechin and epicatechin, depolymerization through thiolysis was carried out by following standard conditions using cysteamine, which was preferred to toluene-*α*-thiol for being more user-friendly and much less toxic [28]. The reaction mixture was analysed by HPLC (Figure 2). In agreement with the MALDI-TOF MS results, the condensed tannins from stem bark and fine root consists primarily of procyanidins. The major product observed was the 4β-(2-aminoethylthio) epicatechin (Cya-EC) along with the small amount of (+)-catechin (Cat), (-)-epicatechin (EC), and 4β-(2-aminoethylthio) catechin (Cya-Cat). This result suggested that there are significant amounts of epicatechin extension units in condensed tannins from stem bark and fine root.

### 2.4. DPPH Radical Scavenging Activity

The relatively stable organic radical DPPH has been widely used in the determination of antioxidant activity of single compounds as well as the different plant extracts [29]. The reduction capability of DPPH radical is determined by the decrease in absorbance at 517 nm induced by antioxidants [30]. Great decreases in a concentration-dependent manner of remaining DPPH indicated that condensed tannins from stem bark and fine root of *C. equisetifolia* possess potent free radical-scavenging activity (Table 3).

**Table 3:** Concentration effect of condensed tannins from stem bark and fine root of *C. equisetifolia *on their free radical scavenging activity (DPPH) and reducing capacity (FRAP) (n = 3).

Concentration (μg/mL)	DPPH *^a^*		FRAP *^b^*	
	Stem bark	Fine root	Stem bark	Fine root
15.63	91.48 ± 0.23d	89.45 ± 0.42d	0.13 ± 0.01a	0.14 ± 0.00a
31.25	83.33 ± 0.78c	80.38 ± 0.84c	0.26 ± 0.01b	0.26 ± 0.00b
62.5	66.42 ± 1.00b	62.73 ± 0.65b	0.51 ± 0.00c	0.53 ± 0.00c
125	39.98 ± 1.08a	31.92 ± 0.09a	0.99 ± 0.00d	1.01 ± 0.01d

*^a^* Data are presented as the percentage of remaining DPPH; *^b^* Data are presented as the absorbance at 593 nm; Different letters on the same column show significant differences from each other at *P* < 0.05; Statistical analysis was done by Duncan’s multiple range tests.

The quality of the antioxidants about the condensed tannins from stem bark and fine root of *C. equisetifolia *was determined by the IC_50_ values (the concentration with scavenging activity of 50%). A lower value of IC_50_ indicates greater antioxidant activity. The IC_50 _values of the stem bark (101.69 ± 2.24 µg/mL) and fine root (89.32 ± 0.21 µg/mL) were significantly lower than the postive compounds ascorbic acid (110.87 ± 0.88 µg/mL) and BHA (115.66 ± 2.13 µg/mL), indicating the condensed tannins of stem bark and fine root exhibited the higher radical scavenging effect than them. The scavenging effect on the DPPH radical decreased in the order: fine root > stem bark > ascorbic acid > BHA (Table 4).

**Table 4:** Antioxidant activities of the condensed tannins from stem bark and fine root of *C. equisetifolia* using the (DPPH) free radical-scavenging assay and the (FRAP) ferric-reducing antioxidant power assay (n = 3).

Samples	Antioxidant activity	
	IC_50_/DPPH (µg/mL) *^a^*	FRAP (mmol AAE/g) *^b^*
Stem bark	101.69 ± 2.24b	5.70 ± 0.03b
Fine root	89.32 ± 0.21a	5.87 ± 0.04c
BHA	115.66 ± 2.13d	5.21 ± 0.04a
Ascorbic acid	110.87 ± 0.88c	--

*^a^* The antioxidant activity was evaluated as the concentration of the test sample required to decrease the absorbance at 517 nm by 50% in comparison to the control; *^b^* FRAP values are expressed in mmol ascorbic acid equivalent/g sample in dry weight; Different letters on the same column show significant differences from each other at *P* < 0.05; Statistical analysis was done by Duncan’s multiple range tests.

### 2.5. Ferric Reducing Antioxidant Power (FRAP)

The FRAP assay is based on the redox reaction of ferric ion in the presence of reducer. The reduction capacity of a compound may serve as a significant indicator of its potential antioxidant activity [31]. A higher absorbance corresponds to a higher ferric reducing power. All tannins showed increased ferric reducing power with the increasing concentration (Table 3). The FRAP value, used to determine the antioxidant ability of stem bark and fine root of *C. equisetifolia* in present study, was expressed in ascorbic acid equivalents. The FRAP values for stem bark and fine root are 5.70±0.03 and 5.87±0.04 mmol AAE/g dried tannins, and were both significantly higher than that of BHA (5.21±0.04 mmol AAE/g dried sample, Table 4).

## 3. Experimental

### 3.1. Chemicals and Materials

All solvents used were of analytical reagent (AR) purity grade. 1,1-Diphenyl-2-picrylhydrazyl (DPPH), 2,4,6-tripyridyl-*s*-triazine (TPTZ), ascorbic acid, butylated hydroxyanisole (BHA), and cesium chloride were purchased from Sigma-Aldrich (USA). Sephadex LH-20 was purchased from Amersham (USA). Stem bark and fine root of *C. equisetifolia* were collected from Chihu Forestry Center of Huian County (23°45′N, 118°55′E), Fujian Province, China. The *C. equisetifolia* plantations were artificial, pure forests planted in 1988, with the coverage of dense forest 0.8, tree density 1535 tree/ha, canopy height 13.5 m and DBH (diameter at breast height) 17.32 ± 2.5 cm. Thirty trees with similar height and growth conditions were chosen and divided into three groups (10 trees in a group). About 50 g/tree stem bark and fine root were collected, washed, freeze-dried (48 h) and then ground to pass through the 40-mesh sieve. The samples were stored at -20 °C prior to analyses.

### 3.2. Extraction and Purification of the Condensed Tannins

Extraction and purification of the condensed tannins were described by Lin *et al.* [32]. Briefly, freeze-dried stem bark and fine root powder (each 25 g) were extracted thrice with 7:3 (v/v) acetone/water solutions at room temperature. Each extract was filtered and pooled, and the solvent was removed under reduced pressure by use a rotary evaporator at 38 °C. The remaining aqueous fraction was extracted thrice with hexane in order to remove chlorophyll and lipophilic compounds. The remaining crude tannin fraction was chromatographed on an LH-20 column (Pharmacia Biotech, Uppsala, Sweden) which was first eluted with methanol-water (50:50, v/v) and then with acetone- water (7:3, v/v). The last fraction purified tannins were freezed-dried and stored at -20 °C before analysis by MALDI-TOF mass spectrometry.

### 3.3. Determination of Total Phenolics and Extractable Condensed Tannins

The amount of total phenolics was determined using the Folin-Ciocalteu method [33]. Briefly, an aliquot of extract (0.2 mL) was added to a 10 mL volumetric flask containing distilled H_2_O (0.3 mL). Folin-Ciocalteu reagent (0.5 mL) and 20% Na_2_CO_3_ solution (2.5 mL) were added to the mixture and shaken. After incubation for 40 min at room temperature, the absorbance *versus* a blank was determined at 725 nm. Total phenolics contents of extracts were expressed as mg tannic acid equivalents (TAE)/g extract. Extractable condensed tannins were assayed by the butanol-HCl method [34], using respective purified condensed tannins as the standards. All samples were analyzed in three replications.

### 3.4. MALDI-TOF MS Analysis

The MALDI-TOF MS spectra were recorded on a Bruker Reflex III instrument (Germany). The irradiation source was a pulsed nitrogen laser with a wavelength of 337 nm, and the duration of the laser pulse was 3 ns. In the positive reflectron mode, an accelerating voltage of 20.0 kV and a reflectron voltage of 23.0 kV were used. 2,5-Dihydroxybenzoic acid (DHB, 10 mg/mL 30% acetone solution) was used as the matrix. The sample solutions (10 mg/mL 30% acetone solution) were mixed with the matrix solution at a volumetric ratio of 1:3. The mixture (1 µL) was spotted to the steel target. Amberlite IRP-64 cation-exchange resins (Sigma-Aldrich, USA), equilibrated in deionized water, and was used to deionize the analyte-matrix solution thrice. Cesium trifluoroacetate (1.52 mg/mL) was mixed with the analyte-matrix solution (1:3, v/v) to promote the formation of a single type of ion adduct ([M+Cs]^+^) [35].

### 3.5. Thiolysis of the Condensed Tannins for HPLC Analysis

Thiolysis was carried out according to the method of Torres and Lozano [36]. A condensed tannin solution (4 mg/mL in methanol) was prepared. A sub-sample (50 µL) was placed in a vial and to this was added hydrochloric acid in methanol (3.3%, v/v; 50 µL) and cysteamine hydrochloride in methanol (50 mg/mL, 100 µL). The solution was heated at 40°C for 30 min, and cooled to room temperature. The size and composition of the condensed tannins were estimated from the RP-HPLC analysis of the depolymerised fractions [28]. Briefly, the terminal flavan-3-ols units were released as such by acid cleavage in the presence of cysteamine whereas the extension moieties were released as the C4 cysteamine derivatives. Thiolysis reaction media (20 µL) filtrated through a membrane filter with an aperture size of 0.45 µm was analyzed by RP-HPLC.

The high performance liquid chromatograph was an Agilent 1200 system (Palo, Alto, CA, USA) equipped with a diode array detector and a quaternary pump. The thiolysis media were further analyzed using LC/MS (QTRAP 3200, USA) with a Hypersil ODS column (4.6 mm × 250 mm, 2.5 μm) (China). The mobile phase was composed of solvent A (0.5% v/v trifluoroacetic acid (TFA) in water) and solvent B (0.5% v/v TFA in acetonitrile). The gradient condition was: 0-5 min, 3% B (isocratic); 5-15 min, 3%-9% B (linear gradient); 15-45 min, 9%-16% B (linear gradient), 45-60 min, 16%-60% B (linear gradient). The column temperature was ambient and the flow-rate was set at 1 mL/min. Detection was at 280 nm and the UV spectra were acquired between 200-600 nm. Degradation products were identified on chromatograms according to their relative retention times and their UV-visible spectra. Each sample analysis was repeated three times.

### 3.6. DPPH Radical Scavenging Activity

The free radical scavenging activities of the purified condensed tannins on the DPPH radical were measured using the method described by Braca *et al.* [37]. A 0.1 mL sample of various concentrations of each freeze-dried purified condensed tannins at different concentration (15.63-250 µg/mL) was added to DPPH solution (3 mL, 0.1 mM in methanolic solution). An equal amount of methanol and DPPH served as control. After the mixture was shaken and left at room temperature for 30 min, the absorbance at 517 nm was measured. Lower absorbance of the reaction mixture indicates higher free radical scavenging activity. The IC_50_ value, defined as the amount of antioxidant necessary to decrease the initial DPPH concentration by 50%, was calculated from the results and used for comparison. The capability to scavenge the DPPH radical was calculated by using the following equation:

DPPH scavenging effect (%) = [(A_1_–A_2_)/A_1_] ×100

where A_1_ = the absorbance of the control reaction; A_2_ = the absorbance in the presence of the sample. BHA and ascorbic acid were used as standards.

### 3.7. Ferric Reducing/Antioxidant Power (FRAP) Assay

FRAP assay is a simple and reliable colorimetric method commonly used for measuring the total antioxidant capacity [38]. In brief, prepared freshly FRAP reagent (3 mL) was mixed with test sample (0.1 mL) or methanol (0.1 mL, for the reagent blank). The FRAP reagent was prepared from 300 mmol/L acetate buffer (pH 3.6), 20 mmol/L ferric chloride and 10 mmol/L TPTZ made up in 40 mmol/L hydrochloric acid. All the above three solutions were mixed together in the ratio of 25:2.5:2.5 (v/v/v). The absorbance of reaction mixture at 593 nm was measured spectrophotometrically after incubation at 25 °C for 10 min. The FRAP values, expressed in mmol ascorbic acid equivalents (AAE)/g dried tannins, were derived from a standard curve.

### 3.8. Statistical Analysis

All data were expressed as means ± standard deviation of three independent determinations. One-way analysis of variance (ANOVA) was used, and the differences were considered to be significant at *P *< 0.05. All statistical analyses were performed with SPSS 13.0 for Windows.

## 4. Conclusions

Structures of condensed tannins from stem bark and fine root of *C. equisetifolia* characterized by MALDI-TOF MS analysis showed that the condensed tannins consist predominantly of procyanidin combined with prodelphinidin and propelargonidin. The HPLC analysis revealed that the epicatechin is the main extension unit of condensed tannins from stem bark and fine root. These condensed tannins had different polymer chain length varying from trimers to tridecamer for stem bark and to pentadecamer for fine root. The condensed tannins extracted from the stem bark and fine root all showed a very good DPPH radical scavenging activity and ferric reducing power, suggesting that these extracts may be considered as new sources of natural antioxidants for food and nutraceutical products.
